# Effects of an actuated ankle exoskeleton on walking stability in healthy adults: a controlled laboratory study

**DOI:** 10.1186/s12984-026-02151-y

**Published:** 2026-09-08

**Authors:** Cagla Kettner, Melina Beyerlein, Charlotte Marquardt, Miha Dežman, Tamim Asfour, Thorsten Stein

**Affiliations:** 1 BioMotion Center, Institute of Sports and Sports Science, Karlsruhe Institute of Technology, 76131 Karlsruhe, Germany; 2 High Performance Humanoid Technologies (H2T), Institute for Anthropomatics and Robotics, Karlsruhe Institute of Technology, 76131 Karlsruhe, Germany

**Keywords:** Motor control, Wearable robotics, Local dynamic stability, Maximum Lyapunov exponent, Detrended fluctuation analysis, Gait variability

## Abstract

**Background:**

Ankle exoskeletons are widely used to reduce the metabolic cost of walking, yet their effects on walking stability during unperturbed gait remain insufficiently understood. Walking stability can be characterized using complementary measures that capture stride-to-stride variability, global temporal organization, and local dynamic stability. Understanding how walking with an actuated ankle exoskeleton system influences these different aspects of gait stability is essential for the safe design and control of wearable robotic devices.

**Methods:**

Eighteen healthy adults walked on a treadmill at a constant speed (1.1 m/s) with and without an actuated bilateral ankle exoskeleton in a randomized crossover design. Spatiotemporal variability was quantified using coefficients of variation (CoV) of stride length, step width, and stance ratio. Global gait stability was assessed using detrended fluctuation analysis of stride time. Local dynamic stability was evaluated using maximum Lyapunov exponent calculated for the trunk, hip, upper leg, lower leg, and foot. Paired-samples two-sided t-tests were used to compare conditions.

**Results:**

Walking with the ankle exoskeleton resulted in increased stride-to-stride spatiotemporal variability, reflected by higher CoV values for stride length (*p* < 0.001) and stance ratio (*p* = 0.005), while mean stride length and step width remained unchanged. Mean stance ratio was reduced in the exoskeleton condition (*p* < 0.001). Global gait stability did not differ between conditions, indicating preserved long-range temporal gait organization. Local dynamic stability increased at the lower leg (*p* < 0.001) and foot (*p* = 0.019) when walking with the exoskeleton.

**Conclusions:**

Walking with the actuated ankle exoskeleton alters gait control across multiple levels during steady walking. While stride-to-stride variability in stride length and stance ratio increased, global gait stability remained unchanged. Local dynamic stability was increased at the lower leg and foot, suggesting segment-specific effects of ankle-level assistance close to the assisted joint. However, these findings should be interpreted as the combined effect of wearing the exoskeleton and receiving active assistance, rather than the isolated effect of plantarflexion assistance. These results provide insight for the design and control of ankle exoskeletons with respect to stability-related effects during walking.

**Supplementary Information:**

The online version contains supplementary material available at https://doi.org/10.1186/s12984-026-02151-y.

## Background

Lower-limb wearable exoskeletons are increasingly used to assist locomotion in rehabilitation, mobility support, industrial ergonomics, and performance augmentation [1–8]. Among these devices, ankle exoskeletons have received particular attention because the ankle plays a central role in forward propulsion, mechanical energy transfer, and balance control during gait [8–15]. Both passive and actuated ankle exoskeletons can reduce the metabolic cost of walking by supporting ankle plantarflexion moments and mechanical work during stance, thereby lowering biological ankle moments and plantarflexor muscle activity while largely preserving ankle joint kinematics [8, 13, 16]. Despite these potential benefits, ankle assistance modifies limb mechanics and may alter sensory feedback and neuromotor control strategies; therefore, its effects on walking stability remain an important question for safe and effective exoskeleton design [17, 18].

Stability during walking has been defined and operationalized using multiple complementary approaches, including linear measures such as the margin of stability, lateral foot placement, and variability-based measures (e.g., coefficient of variation (CoV)) as well as nonlinear measures such as detrended fluctuation analysis (DFA) and local dynamic stability metrics (e.g., maximum Lyapunov exponent (MLE)) [9, 19–24]. In this context, variability refers to fluctuations observed across repeated executions of the same task, such as stride-to-stride variations during walking [25]. Variability-based measures are sometimes used as proxies for stability, based on the notion that increased variability may indicate proximity to the limits of stable locomotion [26, 27]. However, variability does not necessarily reflect reduced stability, as elevated variability can also represent functional adaptability and flexible neuromotor control rather than instability [25, 28]. Consequently, stability-related outcomes, particularly those based on variability, must be interpreted with caution when evaluating externally assisted walking.

Despite the growing number of studies on ankle exoskeleton energetics and mechanics, relatively few studies have explicitly quantified walking stability [8, 13, 14, 16, 18, 29]. To date, only limited experimental evidence exists on stability-related metrics during unperturbed walking with ankle exoskeleton assistance [15, 30]. The available studies suggest that ankle assistance does not have a uniform effect on gait stability, but may influence different stability-related outcomes depending on the assistance timing, power magnitude, controller design, and the stability construct assessed. For example, Antonellis et al. [30] reported that specific combinations of assistance timing and power could increase local dynamic stability during unperturbed treadmill walking, while other stability measures showed opposing trends. Similarly, Choi et al. [15] demonstrated that appropriately designed ankle assistance could preserve or improve local dynamic stability during unperturbed walking, with effects varying across control strategies and stability measures. These findings highlight that walking stability cannot be sufficiently characterized by a single metric. This motivates the use of complementary analytical approaches to assess different levels of gait control, including CoV to quantify spatiotemporal variability, DFA to assess global temporal gait organization, and MLE to evaluate local dynamic stability.

Advanced nonlinear methods, such as DFA of stride time and MLE of body segments, provide complementary insights into gait dynamics. Specifically, DFA captures long-range temporal correlations, reflecting global gait organization, while MLE quantifies local dynamic stability, indicating sensitivity to small perturbations. Both measures are sensitive to subtle changes in gait organization induced by external assistance [19, 20, 22, 25]. In this context, increased long-range correlations (e.g. higher DFA-α values) are interpreted as reduced system adaptability and, consequently, lower global stability [31, 32]. When combined with traditional linear measures, such as the CoV, these nonlinear approaches enable a more comprehensive characterization of walking stability across spatiotemporal variability, global gait organization, and local segment dynamics.

In summary, while ankle exoskeletons are well established as effective devices for reducing the metabolic cost of walking [8, 16, 33], their effects on walking stability during unperturbed walking in healthy adults have not yet been sufficiently understood. Addressing this gap is relevant for the stability-related evaluation of ankle exoskeletons, particularly for future rehabilitation and daily-life assistance applications where stable gait support is essential. The purpose of this study is therefore to investigate how walking with an actuated ankle exoskeleton system affects walking stability in healthy adults during steady, unperturbed walking. Although healthy adults were studied to establish baseline biomechanical and neuromotor-control effects, examining DFA and MLE during walking with the exoskeleton may indicate whether this specific exoskeleton condition preserves global temporal gait organization and alters segment-specific local dynamic stability. Such information may provide a basis for future studies that systematically vary assistance parameters, such as timing, magnitude, or duration, to investigate their specific effects on gait stability. This may be relevant for future applications in mobility-impaired populations, such as individuals after stroke, for whom exoskeleton control should support both stable and adaptable gait. On this basis, three hypotheses were formulated. First, it was hypothesized that walking with the actuated ankle exoskeleton system would alter stride-to-stride spatiotemporal variability, reflected by differences in CoV values, because plantarflexion assistance may reduce biological muscular contributions, alter sensory feedback, and increase reliance on the device, while device-related mechanical constraints and adaptation of gait control during early exposure may further increase step-to-step variability [3, 17, 34–38]. Second, it was hypothesized that global stability, assessed using DFA of stride time, would remain unchanged, as long-range temporal gait organization is likely preserved during steady, unperturbed walking [32, 39]. Third, it was expected that walking with the exoskeleton would alter local dynamic stability of lower-body segments close to the assisted ankle joint, as quantified by MLE [30], whereas more proximal segments such as the hip and trunk were expected to be less affected [40].

## Methods

### Participants

The required sample size was determined a priori based on local stability results reported by Choi et al. [15] using a one-sided paired t-test (α = 0.05, power = 0.90). The power analysis indicated a required sample size of 17 participants. Eighteen participants (female *n* = 6, age: 24.9 ± 2.9 years; height: 1.79 ± 0.06 m; body mass: 70.8 ± 9.0 kg) were ultimately included in the analysis to allow for a balanced crossover design with equal ordering of the exoskeleton (EXO) and no-exoskeleton conditions (noEXO). Because the final statistical analyses were revised to two-sided paired-samples t-tests, the adequacy of the included sample was additionally verified using a two-sided paired t-test with α = 0.05 and 80% power. This sensitivity check indicated that 16 participants would be required, thereby supporting the adequacy of the included sample size. All participants were free of neurological or musculoskeletal impairments. They were informed about the purpose and procedures of the study and provided written informed consent prior to participation. The study protocol was approved by the ethics committee of the Karlsruhe Institute of Technology (KIT).

### Exoskeleton

**Fig. 1:**
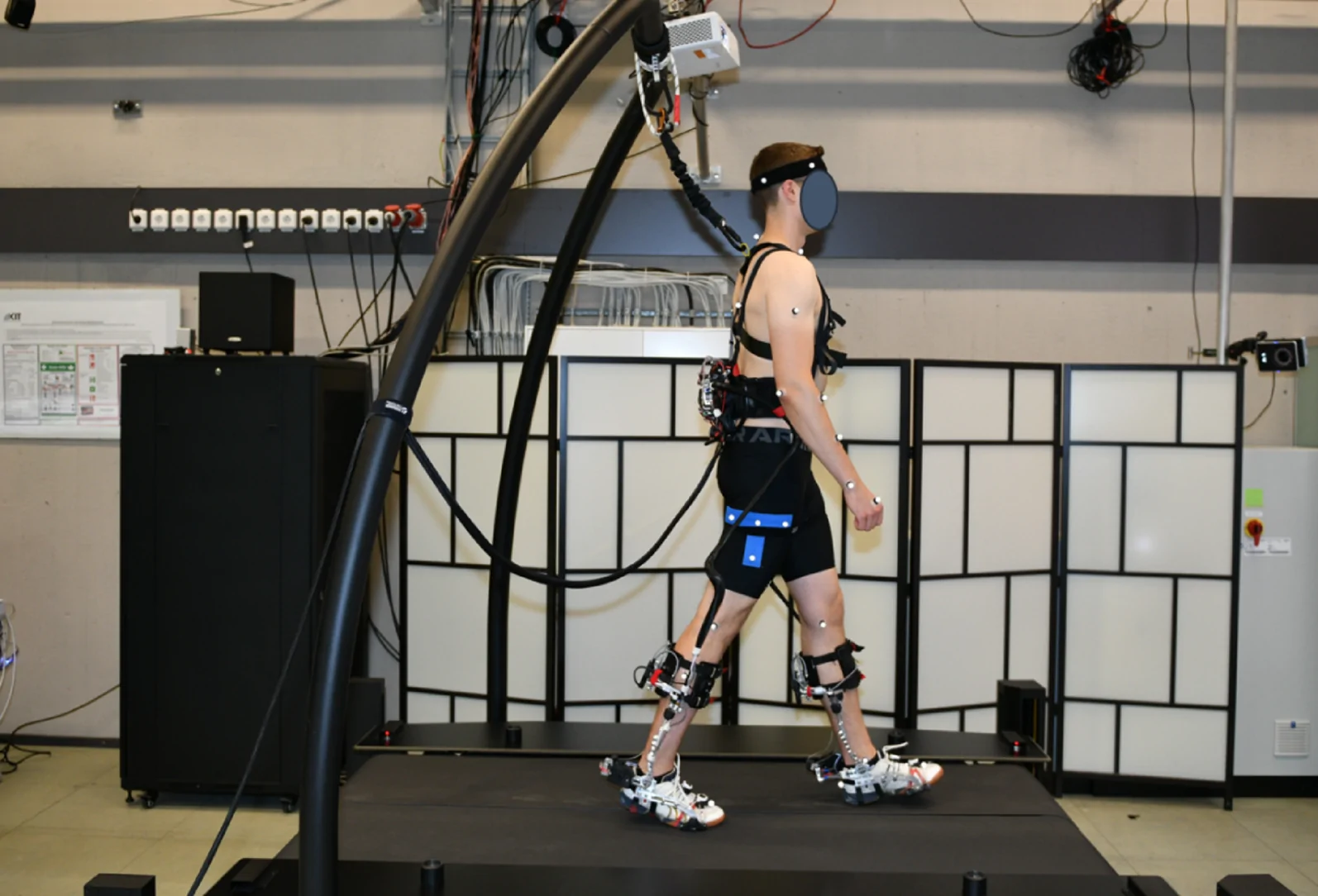
Experimental setup showing a participant on the split-belt treadmill wearing the ankle exoskeleton and reflective motion-capture markers

The ankle exoskeleton used in this study was an adapted version of a previously described device [41]. It consisted of a rigid frame with shank and foot segments connected by a parallelogram linkage and multiple joints, allowing all three ankle degrees of freedom (Fig. 1). Inversion/eversion, internal/external rotation, and dorsiflexion were implemented as passive degrees of freedom, whereas plantarflexion was actively actuated through a symmetrical parallel joint architecture [41, 42]. The exoskeleton was adjustable to accommodate common shoe sizes (EU 38–46), varying shoe widths, and different calf circumferences. The shoes were secured using a cable tensioning system and under-shoe straps, providing a stable fit without restricting sole flexibility. The frontal ankle joint axis of rotation was adjustable to improve anatomical alignment. A centrally mounted actuation unit was secured via an individually adjustable hip belt positioned close to the body’s center of mass to optimize power transmission and user comfort. The bilateral ankle exoskeleton weighed 2.4 kg (1.2 kg per side), while the central actuation unit weighed 3.3 kg. Torque assistance was provided via cable-driven actuation during push-off (terminal stance and pre-swing) to support ankle plantarflexion using an adjustable torque profile (Supplementary Figure S1). The torque profiles were derived from the optimal assistance profiles reported by Franks et al. [43], and were generated using two cubic Hermite splines. The profile was defined by peak torque, peak time, rise time, fall time, and preload. The target peak assistance torque was initially set to 20 Nm at the ankle joint and was not individualized to body mass. However, four of the 18 participants received a lower applied peak torque, either due to technical issues, such as unintended triggering of the heel switch by the exoskeleton assistance, or because the participant could not comfortably adapt to the higher assistive torque. Across participants, the resulting applied peak torque was 16.7 ± 5.8 Nm, corresponding to 0.24 ± 0.09 Nm/kg when normalized to body mass. Peak time was 48.7 ± 2.2% of the gait cycle, rise time was 21.1 ± 7.1%, fall time was 13.9 ± 9.9%, and preload was 0.5 ± 0.2 Nm. The timing of the torque profile was determined from a linear estimate of the gait phase calculated from the duration and gait events of the last five strides, and thus automatically individualized for each participant. Gait events such as heel strike and toe-off were detected using force-sensitive resistors embedded in an insole under the heel and toe.

### Study design

This experiment constituted a subset of a larger experimental protocol; only the procedures relevant to the present study are described here. The experimental timeline is shown in Supplementary Figure S2. Following an initial fitting of the ankle exoskeleton, participants completed a standardized warm-up on a split-belt treadmill (M-Gait, Motek Medical, Amsterdam, The Netherlands). During all treadmill walking trials, participants were secured with an overhead safety harness to prevent falls while not providing body-weight support. The warm-up consisted of 5 min of walking at a constant speed of 1.1 m/s without the exoskeleton. After the warm-up, participants were randomly assigned to one of two condition orders in a counterbalanced crossover design. Half of the participants (*n* = 9) began with the noEXO condition and walked for 3 min; this trial was used for subsequent analysis. Participants then donned the ankle exoskeleton and completed a 10-min adaptation period while walking [38], followed by a 3-min walking trial that was analyzed. The remaining participants (*n* = 9) began with the EXO condition. After the warm-up, they donned the exoskeleton and walked for 10 min to allow adaptation [38], followed by a 3-min walking trial that was included in the analysis. Participants then took a 5-min break, after which they walked for 5 min without the exoskeleton to allow deadaptation. This was followed by an additional 3-min break, and finally a 3-min noEXO walking trial, which was analyzed. Across all conditions, treadmill speed was kept constant at 1.1 m/s [44].

### Data acquisition and preprocessing

Three-dimensional whole-body kinematics were recorded using a motion capture system (Vicon Motion Systems, Oxford, UK) comprising 16 cameras, sampling at 200 Hz. A total of 49 reflective markers were placed on anatomical landmarks of the head, trunk, pelvis, and upper and lower extremities. In addition, 12 cluster markers were attached to the thigh and shank segments to reduce the influence of soft-tissue artifacts [45]. Marker trajectories were reconstructed offline using Vicon Nexus (V2.15). Subsequent data processing and analyses were performed in MATLAB (R2024a; MathWorks Inc., Natick, MA, USA). Marker trajectories were low-pass filtered using a fourth-order Butterworth filter with a cutoff frequency of 10 Hz.

### Data analysis

Because walking stability cannot be fully characterized by a single outcome measure, the analysis was designed to quantify complementary aspects of gait control. CoV was used to assess the magnitude of stride-to-stride spatiotemporal variability [25, 26], DFA to assess the global temporal organization of stride timing [32, 39], and MLE to quantify local segment-level dynamic stability [25, 46]. This combined approach was selected to compare how gait stability differed between walking with and without the actuated ankle exoskeleton system.

#### Spatiotemporal variability

Eighty strides occurring after the first 30 s of walking were analyzed. Gait events of both legs were used to define strides and to determine stance phase. Initial contact and toe-off events were identified from sagittal-plane kinematic data using a marker-based approach. Initial contact was defined as the instant of maximal anterior displacement of the heel marker relative to the pelvis, whereas toe-off was defined as the instant of maximal posterior displacement of the toe marker relative to the pelvis [47]. All detected gait events were visually verified using stick-figure representations. Stride length, step width, and stance ratio normalized to stride time derived from 80 strides (160 steps) were used to compute participant-specific mean values and CoV. Corresponding mean values were additionally reported to complement the spatiotemporal variability analysis and to facilitate interpretation of the data.

#### Global stability

DFA was applied to quantify long-range correlations in stride time variability [22, 32]. A total of 120 consecutive strides were included in the analysis. The stride time series, $\:{x}$, was first mean-centered and then integrated by cumulative summation (Eq. 1). This integration step converts stride-to-stride deviations from the mean into a cumulative profile, allowing fluctuations in stride timing to be evaluated across different time scales. The resulting signal, $\:{A}\left({w}\right)$, was divided into non-overlapping windows with lengths ranging from $\:\boldsymbol{\Delta\:}\mathbf{n}\:$= 4 to 60 samples. This window range was selected in accordance with recommendations for short time series (≤ 128 strides) [48]. Within each window, a second-order polynomial was fitted and subtracted to remove low-frequency trends. The root-mean-square fluctuation of the detrended signal was then computed for each window size (Eq. 2; [49]). Thus, F(n) represents the root-mean-square magnitude of the residual fluctuations after local trends have been removed within each window size.

1 $$\:A\left(w\right)=\:\sum\:_{i=1}^{w}(x\left(i\right)-{x}_{avg})$$

2 $$\:F\left(\varDelta\:\mathrm{n}\right)=\:\sqrt{\frac{1}{N}\:\sum\:_{w=1}^{N}{(A\left(w\right)-{A}_{\varDelta\:\mathrm{n}}(w\left)\right)}^{2}}$$

If scale-free dynamics are present, the relationship between the fluctuation magnitude $\:{F}\left({\varDelta\:}{n}\right)$ and window size $\:{\varDelta\:}{n}$ follows a power-law and appears linear on a log–log scale (Eq. 3). The DFA-α value was therefore obtained as the slope of the linear relationship between log F(n) and log n. The slope of this relationship (DFA-α) characterizes the strength of temporal correlations, with values between 0.5 and 1 indicating persistent correlations [22, 50]. Within this range, higher DFA-α values reflect reduced global gait stability, whereas lower values indicate greater stability.

3 $$\:\mathrm{F}\:(\varDelta\:\mathrm{n})\:\propto\:\:{(\varDelta\:\mathrm{n})}^{\mathrm{D}\mathrm{F}\mathrm{A}-{\upalpha\:}}$$

#### Local stability

Local dynamic stability was assessed for the trunk, hip, upper leg, lower leg, and foot using the MLE derived from the vertical displacement time series of marker clusters comprising three to four markers per body segment [23, 46]. Marker-based time series, $\:{z}\left({t}\right)$, spanning 120 consecutive strides were time-normalized to 10,000 samples [51]. The embedding dimension ($\:{m}$) was identified using the false nearest neighbor approach, with the maximum value observed across all trials selected for each segment ($\:{m}$_trunk_ = 8, $\:{m}$_hip_ = 8, $\:{m}$_upper leg_ = 10, $\:{m}$_lower leg_ = 10, and $\:{m}$_foot_ = 10). The time delay (τ) was determined from the first local minimum of the average mutual information function, and the median τ across all trials was used for each segment (τ_trunk_ = 39, τ_hip_ = 39, τ_upper leg_ = 36, τ_lower leg_ = 73, and τ_foot_ = 40). The MLE was computed using Rosenstein’s algorithm (Eq. 4) [52]. The short-term divergence rate (λ) was calculated as the slope of the average logarithmic divergence curve over 0–0.5 stride cycles, representing the rate at which initially neighboring trajectories in the reconstructed state space diverged over time. Lower λ values indicate slower divergence and therefore greater local dynamic stability, whereas higher λ values indicate faster divergence and lower local dynamic stability.

4 $$\:\mathrm{S}\left(\mathrm{t}\right)=\left[z\left(t\right),\:z\left(t+\tau\:\right),\dots\:,\:z\left(t+\left(m-1\right)\:\tau\:\right)\:\right]$$

### Statistics

Statistical analyses were performed using SPSS (version 29.0; IBM Corp., Armonk, NY, USA) on the mean and CoV of stride length, step width, and stance ratio, as well as DFA-α and the λ of the trunk, hip, upper leg, lower leg, and foot. Stride length, stance ratio, and λ values for the upper leg, lower leg, and foot were evaluated separately for the left and right legs. As the results showed identical patterns of statistical significance, and no obvious asymmetry was observed during the measurements, only the data from the left leg are presented for clarity. Data normality was assessed using the Kolmogorov–Smirnov test. Differences between the EXO and noEXO conditions were evaluated using paired-samples two-sided t-tests. Effect sizes were quantified using Cohen’s d and interpreted as small (≤ 0.50), medium (0.50–0.80), or large (≥ 0.80) [53]. Statistical significance was defined at an alpha level of 0.05.

## Results

### Spatiotemporal variability

The spatiotemporal gait outcomes are summarized in Table 1, the corresponding 95% confidence intervals are provided in Supplementary Table S1, and violin plots with individual participant data are shown in Supplementary Figure S3. Among the mean spatiotemporal variables, only stance ratio differed significantly between the EXO and noEXO conditions (*p* < 0.001, d = −1.54). Specifically, stance ratio was reduced during walking with the ankle exoskeleton, indicating shorter ground contact time relative to stride time. In contrast, the CoV of stride length (*p* < 0.001, d = 1.20) and stance ratio (*p* = 0.005, d = 0.77) were higher in the EXO condition compared with noEXO, indicating increased spatiotemporal variability when walking with the exoskeleton.

**Table 1:** Mean values and coefficients of variation (CoV) of stride length, step width, and stance ratio during walking with (EXO) and without (noEXO) the ankle exoskeleton. Descriptive statistics are presented as mean ± standard deviation. Statistically significant results are indicated in bold

	EXO	noEXO	*p*	Cohen’s d
Mean stride length (m)	1.32 ± 0.09	1.32 ± 0.08	0.924	0.02
Mean step width (m)	0.15 ± 0.03	0.14 ± 0.03	0.273	0.27
Mean stance ratio (%)	66.07 ± 0.96	67.27 ± 0.62	**< 0.001**	**−1.54**
CoV stride length (%)	2.83 ± 1.00	1.87 ± 0.38	**< 0.001**	**1.20**
CoV step width (%)	15.25 ± 3.91	13.18 ± 5.82	0.074	0.45
CoV stance ratio (%)	1.23 ± 0.36	0.97 ± 0.19	**0.005**	**0.77**

### Global stability

The results of the global stability measure (DFA-α) are presented in Table 2, with corresponding 95% confidence intervals provided in Supplementary Table S1 and violin plots with individual participant data shown in Supplementary Figure S4. DFA-α values ranged between 0.5 and 1, indicating persistent, self-similar stride time fluctuations, and did not differ between the EXO and noEXO conditions.

**Table 2:** Global (DFA-α) and local dynamic stability (λ) measures during walking with (EXO) and without (noEXO) the ankle exoskeleton. Descriptive statistics are presented as mean ± standard deviation. Statistically significant results are indicated in bold

	EXO	noEXO	*p*	Cohen’s d
DFA-α	0.66 ± 0.13	0.65 ± 0.14	0.666	0.10
λ_trunk_	0.78 ± 0.06	0.78 ± 0.07	0.948	0.15
λ_hip_	0.70 ± 0.08	0.75 ± 0.07	0.076	−0.45
λ_upper leg_	0.66 ± 0.05	0.67 ± 0.04	0.179	−0.22
λ_lower leg_	0.27 ± 0.05	0.33 ± 0.05	**< 0.001**	**−1.47**
λ_foot_	0.72 ± 0.07	0.76 ± 0.07	**0.019**	**−0.61**

### Local stability

**Fig. 2:**
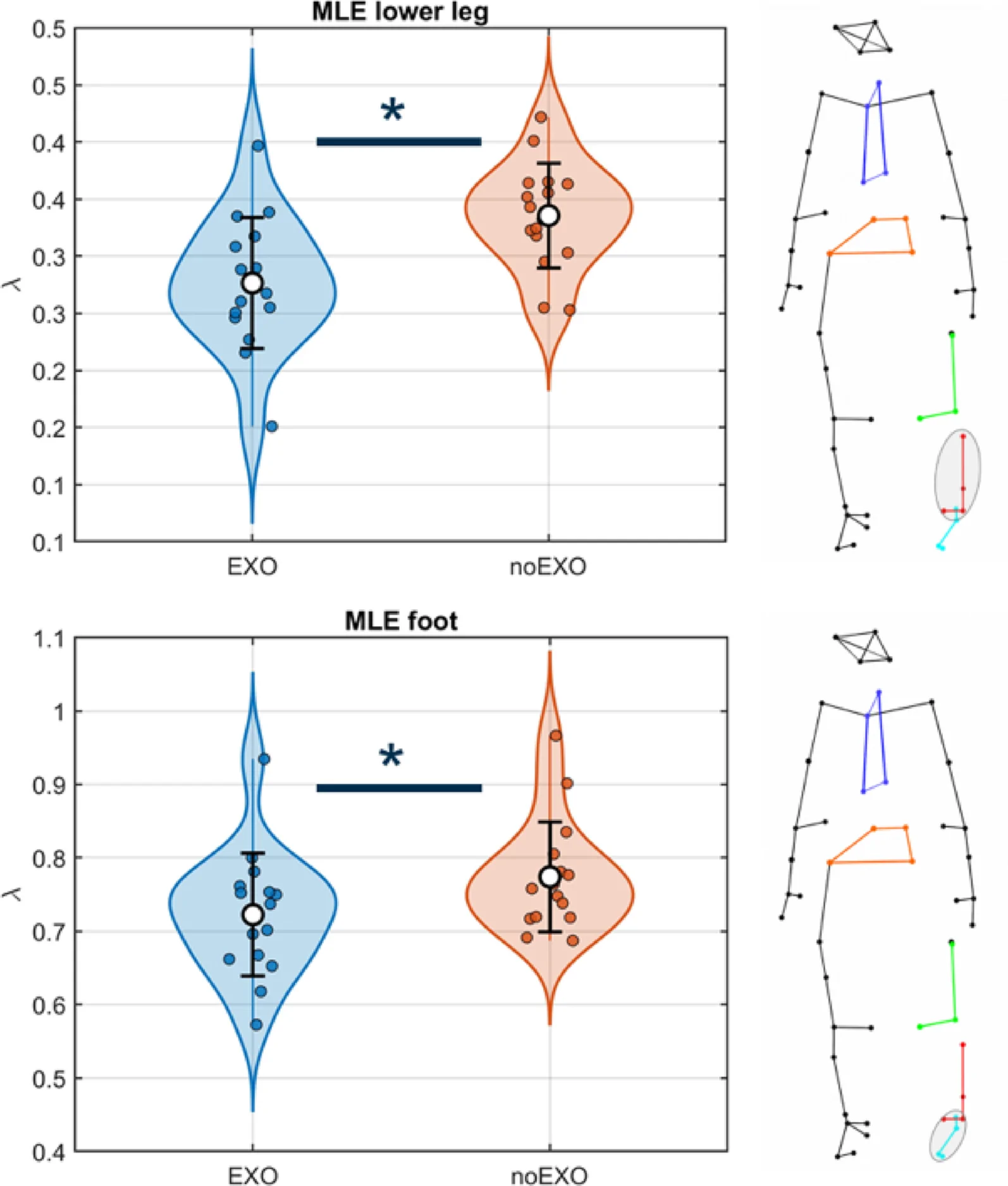
Local dynamic stability quantified by the λ at the lower leg and foot for the exoskeleton (EXO) and no-exoskeleton (noEXO) conditions. Violin plots show the distribution of participant-specific λ values, with individual data points overlaid. Open circles indicate group means, and error bars represent standard deviations. Asterisks (*) denote significant differences between conditions, with EXO showing reduced λ (i.e., increased local stability) at these segments. The stick-figure visualizations on the right illustrate the corresponding body segments used for local stability analysis

The results of the local stability measure (λ) are shown in Table 2, with corresponding 95% confidence intervals provided in Supplementary Table S1. λ values of lower leg and foot were reduced in the EXO condition (lower leg: *p* < 0.001, d = −1.47; foot: *p* = 0.019, d = −0.61), reflecting increased local dynamic stability at the lower leg and foot, respectively. Detailed visualizations of the body segments showing significant differences between the EXO and noEXO conditions are shown in Fig. 2, while visualizations of the body segments without significant differences are provided in Supplementary Figure S4.

## Discussion

This study aimed to investigate the effects of an actuated ankle exoskeleton on spatiotemporal variability, global gait stability, and local dynamic stability during steady, unperturbed walking in healthy adults. In line with the first hypothesis, walking with the exoskeleton resulted in increased stride-to-stride spatiotemporal variability, reflected by higher CoV of stride length and stance ratio, while mean spatiotemporal parameters were largely preserved. Supporting the second hypothesis, global gait stability assessed by DFA remained unchanged between the EXO and noEXO conditions, indicating preserved long-range temporal gait organization. Local dynamic stability increased at the lower leg and foot, which was consistent with the third hypothesis.

### Increased spatiotemporal variability and reduced stance ratio with ankle exoskeleton

Consistent with the first hypothesis, variability of stride length and stance ratio increased when participants walked with the ankle exoskeleton, despite unchanged mean stride length and step width and a decrease in stance ratio. While mean stride length and step width were preserved, the increased variability in stride length and stance ratio suggests that the exoskeleton altered stride-to-stride control without modifying the overall gait pattern. This finding should be interpreted cautiously, as increased variability can reflect neuromotor noise or reduced stability, but may also represent flexible neuromotor control under altered task constraints [25, 28]. However, the present study did not include coordination-based analyses, such as the uncontrolled manifold approach [54, 55]. Therefore, it cannot be determined whether the increased CoV reflected variability that impaired task-level control, variability that reflected functional flexibility, or a combination of both.

In this study, participants were given a 10-min familiarization period prior to data collection, consistent with previous literature showing that most kinematic and muscular adaptation occurs rapidly and that mean gait parameters and energetic outcomes typically stabilize within ~10 min (∼880 steps) of assisted walking [38]. However, because longer exposure was not assessed, it remains unknown whether the increased variability would decrease, persist, or further change with extended usage [36, 37].

In addition to increased variability, stance ratio was reduced when walking with the ankle exoskeleton. A shorter stance phase is biomechanically plausible, as the exoskeleton provided active plantarflexion assistance during late stance, which may have assisted push-off and contributed to an earlier transition from stance to swing [11, 16, 35]. Previous studies have shown that ankle exoskeleton assistance can reduce biological plantarflexor moment and mechanical work during push-off, which can support forward propulsion of the body [13, 35]. In the present study, the active assistance may have reduced the time required to generate sufficient forward and upward momentum for toe-off, resulting in an earlier termination of stance [10, 11, 16, 35]. Importantly, the observed reduction in stance ratio occurred without changes in mean stride length or step width, suggesting that walking with the actuated ankle exoskeleton system influenced the temporal distribution within the gait cycle rather than mean spatiotemporal characteristics of gait. However, this interpretation is specific to the torque profile used in the present study, and different assistance timing parameters, such as later onset, peak torque, or offset timing, may lead to different stance-phase adaptations. Furthermore, because the EXO condition also included the added mass and inertia of the device, this effect should be interpreted as the net response to walking with the actuated exoskeleton system rather than as the isolated effect of plantarflexion assistance.

### Preserved global stability of stride timing during walking with ankle exoskeleton

Supporting the second hypothesis, DFA-α of stride time did not differ between the EXO and noEXO conditions, indicating that global gait stability, as assessed by the long-range temporal structure of stride time, was preserved. This finding is consistent with the interpretation that DFA reflects the global temporal organization of stride time, which is distinct from the magnitude of stride-to-stride variability [22, 32, 50]. In particular, DFA-α reflects temporal correlations across many strides rather than the magnitude of variability [19, 22]. One practical interpretation in the context of exoskeleton use is that participants adapted locally to the exoskeleton (e.g., adjusting push-off as reflected by decreased stance ratio), while the long-range temporal organization of stride time remained unchanged [13, 22, 35]. This interpretation is supported by previous ankle exoskeleton work showing that DFA of temporal gait parameters can remain unchanged under assistance, despite alterations in other aspects of gait control. Specifically, Canete et al. reported that DFA of step time was preserved during self-paced walking with an ankle exoskeleton, while DFA of step width changed [56]. Together, these findings indicate that walking with an ankle exoskeleton does not necessarily modulate global gait stability, as reflected by the long-range temporal organization of stride time, even when spatiotemporal variability and local dynamic stability are altered.

### Improved local dynamic stability at distal segments

Consistent with the third hypothesis, local dynamic stability increased at the lower leg and foot when walking with the ankle exoskeleton, as reflected by reduced MLE values at both segments. This finding aligns with previous ankle exoskeleton work showing that assistance can influence local stability in distal lower-limb segments near the assisted ankle joint [30]. The large effect observed at the lower leg suggests that walking with the actuated ankle exoskeleton system particularly affected lower-leg motion close to the ankle, resulting in increased local dynamic stability at this segment. The moderate increase in local dynamic stability at the foot may be specifically relevant for maintaining stable foot–ground interaction when responding to small perturbations during walking [10, 19, 24].

Consistent with the third hypothesis, local dynamic stability at the more proximal segments was not significantly affected by the exoskeleton. These findings contrast with previous work indicating that proximal segments, particularly the hip and trunk, are sensitive to changes in whole-body balance and dynamic stability, even when mechanical assistance is applied distally [15, 17]. Differences from previous findings may be related to differences in device design and control strategy. For example, Choi et al. [15] reported increased trunk stability during walking with ankle exoskeleton assistance; however, their device used pneumatic artificial muscles with force-based assistance and did not report a directly comparable ankle torque profile. In contrast, in the present study, the stabilizing effects were limited to the lower leg and foot. It should also be noted that the present exoskeleton includes a waist-mounted actuation unit, and the additional mass carried near the trunk may have influenced proximal segment dynamics [3, 57]. This potential mechanical contribution should be considered when interpreting the absence of significant changes in hip and trunk local dynamic stability.

Taken together, these results partially support the third hypothesis by showing increased local dynamic stability at segments close to the assisted ankle joint during walking with the actuated exoskeleton. The absence of significant changes at the upper leg, hip, and trunk indicates that these stabilizing effects did not propagate uniformly across all body segments, highlighting a segment-specific response to walking with the actuated ankle exoskeleton. From a design and control perspective, these findings should not be interpreted as direct evidence for how specific assistance parameters should be tuned, because only one predefined torque profile was tested. Instead, the results suggest that future stability-related evaluations of ankle exoskeletons should combine complementary outcomes across different levels of gait control. In particular, distal local dynamic stability may be relevant to monitor, as the strongest effects were observed close to the ankle, while unchanged DFA-α indicated preserved global temporal gait organization. Future studies should systematically vary assistance magnitude, onset timing, and duration to determine how these parameters affect local stability, stride-to-stride variability, and global gait organization.

### Limitations

Several limitations should be considered when interpreting the results of this study. First, walking speed was fixed for all participants during the experimental trials. Walking speed is known to influence spatiotemporal variability and stability measures, and allowing self-selected or self-paced walking could lead to different adaptation strategies [50, 58–60]. However, fixed-speed treadmill walking is common in laboratory-based gait studies, as it provides controlled and repeatable conditions and allows comparisons across experimental conditions [60, 61]. Moreover, fixed speed reduces confounding effects related to speed-dependent changes in gait dynamics, which was important for comparing the EXO and noEXO conditions under controlled walking conditions.

Second, the mass of the ankle exoskeleton may have influenced gait dynamics independently of active assistance, as device mass and inertia are known to affect gait behavior in ankle exoskeleton studies [3]. Although the exoskeleton was actuated, purely mechanical effects related to added mass and inertia at the distal limb cannot be fully excluded. However, the bilateral ankle components weighed 1.2 kg per side, which is within the lower range reported for ankle exoskeleton devices in the literature, yet may still influence gait mechanics [16, 62]. In addition, the waist-mounted actuation unit resulted in additional mass near the trunk, which may have affected proximal segment dynamics and whole-body motion [57, 63]. Importantly, the present study did not include a zero-torque or transparent exoskeleton condition. Therefore, the observed changes in gait stability and variability cannot be attributed solely to active plantarflexion assistance, but should instead be interpreted as the combined effect of walking with the actuated exoskeleton system, including both the added mechanical load and the assistance provided by the device. Future studies should include no-exoskeleton, zero-torque, and active-assistance conditions to determine the specific contribution of exoskeleton actuation to gait stability and variability.

Third, the estimation of both DFA and MLE was based on time series of 120 strides. For DFA, previous methodological work has shown that estimates are affected by time-series length, but that short stride-interval time series can be used for condition comparisons when appropriate parameters are selected; sample lengths around *N* = 128 have been suggested as acceptable [48]. For MLE, the use of 120 strides was within the range commonly used in gait local dynamic stability analyses [46, 51]. Nevertheless, longer recordings may improve the reliability of both DFA and MLE estimates, particularly for characterizing long-range temporal correlations. In addition, the 10-min familiarization period was consistent with previous work showing that many mean gait and energetic parameters stabilize within approximately 10 min of assisted walking [38]. However, nonlinear variables may follow different adaptation time courses than mean gait or energetic variables.

Finally, the study was conducted in healthy young adults during steady, unperturbed treadmill walking. This allowed for a controlled investigation of stability-related gait dynamics during walking with the exoskeleton, which represents an important basis for understanding fundamental human–exoskeleton interaction. However, the findings should not be interpreted as direct evidence of reactive stability, perturbation recovery, or fall resistance. Responses to perturbations may depend on the type, direction, timing, and magnitude of the perturbation, as well as on task-specific recovery strategies [64]. Therefore, the present results may not directly generalize to perturbed walking or clinical populations. Future work should investigate whether similar stability effects are observed in populations with impaired gait stability and during perturbation-based walking scenarios.

## Conclusions

In conclusion, this study investigated how an actuated ankle exoskeleton affects spatiotemporal variability, global gait stability, and local dynamic stability during steady, unperturbed walking in healthy adults. Walking with the exoskeleton increased stride-to-stride spatiotemporal variability in stride length and stance ratio, while preserving mean stride length and step width, which may reflect altered stride-to-stride control under the tested exoskeleton condition. In addition, stance ratio was reduced, which may be related to the exoskeleton providing plantarflexion assistance during terminal stance and thereby facilitating propulsion.

Global gait stability, assessed with the long-range temporal structure of stride time, remained unchanged, indicating that walking with the actuated ankle exoskeleton did not alter the overall temporal organization of gait. At the local level, walking with the actuated ankle exoskeleton increased local dynamic stability at the lower leg and foot. In contrast, local dynamic stability at more proximal segments, including the upper leg, hip, and trunk, did not change significantly. Together, these findings highlight a segment-specific response to walking with the actuated ankle exoskeleton, characterized by local stabilization close to the ankle, while preserving global gait stability.

Overall, these findings suggest that walking with an actuated ankle exoskeleton system increased stride-to-stride variability in stride length and stance ratio and improved local dynamic stability of the lower leg and foot, while global gait stability remained unchanged. However, because the present study did not include a zero-torque control condition, these effects should be interpreted as the combined effects of wearing the exoskeleton and receiving active assistance, rather than as the isolated effect of plantarflexion assistance. These insights contribute to a more comprehensive understanding of how ankle exoskeletons interact with human gait.

## Supplementary Information

Supplementary Material 1.

## Data Availability

Data for this study are available from the corresponding author on reasonable request.

## Abbreviations

CoV: Coefficient of variation
DFA: Detrended fluctuation analysis
DFA-α: Scaling exponent of detrended fluctuation analysis
EXO: With exoskeleton assistance condition
noEXO: Without exoskeleton condition
MLE: Maximum Lyapunov exponent
λ: Short-term divergence exponent derived from the maximum Lyapunov exponent analysis
