# Supplementary material for: Effects of an actuated ankle exoskeleton on walking stability in healthy adults: a controlled laboratory study
Source: J Neuroeng Rehabil. 2026 Sep 8;23:261. doi: 10.1186/s12984-026-02151-y (PMC13555940; doi:10.1186/s12984-026-02151-y)
Supplement: Supplementary file 1 [file 12984_2026_2151_MOESM1_ESM.docx]

**Supplementary Figure S1:** Applied ankle exoskeleton torque profile over the normalized gait cycle. The solid line shows the group mean, and the shaded area represents the standard deviation across participants. A small preload was added to the assistance profile to reduce slack in the cable transmission. Assistance was deactivated when the forefoot switch was released, ensuring that no assistive torque was applied after toe-off.

**Supplementary Figure S2:** Experimental timeline of the protocol relevant to the present study. Group 1 completed the no-exoskeleton (noEXO) condition before the exoskeleton (EXO) condition, whereas Group 2 completed the EXO condition before the noEXO condition.

**Supplementary Figure S3:** Spatiotemporal gait outcomes during walking with (EXO) and without (noEXO) the ankle exoskeleton. Violin plots show the distribution of participant-specific values for mean stride length (1a), coefficient of variation (CoV) of stride length (1b), mean step width (2a), CoV of step width (2b), mean stance ratio (3a), and CoV of stance ratio (3b). Individual data points are overlaid. Open circles indicate group means, and error bars represent standard deviations. Asterisks (*) denote significant differences between conditions.

| **(1a)** |  | **(1b)** |  |
| --- | --- | --- | --- |
| **(2a)** |  | **(2b)** |  |
| **(3a)** |  | **(3b)** |  |

**Supplementary Figure S4:** Global and local dynamic stability outcomes during walking with (EXO) and without (noEXO) the ankle exoskeleton. Violin plots show the distribution of participant-specific values for DFA-α (1a), λ_trunk_ (1b), λ_hip_ (2a), and λ_upper leg_ (2b). Individual data points are overlaid. Open circles indicate group means, and error bars represent standard deviations.

| **(1a)** |  | **(1b)** |  |
| --- | --- | --- | --- |
| **(2a)** |  | **(2b)** |  |

**Supplementary Table S1**. 95% confidence intervals for mean values and coefficients of variation (CoV) of stride length, step width, and stance ratio, as well as global (DFA-α) and local dynamic stability (λ) measures during walking with (EXO) and without (noEXO) the ankle exoskeleton. Confidence intervals are presented as lower and upper limits.

|  | EXO | noEXO |
| --- | --- | --- |
| Mean stride length (m) | [1.28, 1.37] | [1.28, 1.36] |
| Mean step width (m) | [0.13, 0.16] | [0.13, 0.16] |
| Mean stance ratio (%) | [65.59, 66.54] | [66.97, 67.59] |
| CoV stride length (%) | [2.33, 3.33] | [1.68, 2.06] |
| CoV step width (%) | [13.31, 17.20] | [10.28, 16.08] |
| CoV stance ratio (%) | [1.05, 1.42] | [0.88, 1.07] |
| DFA-α | [0.60, 0.73] | [0.58, 0.72] |
| λ_trunk_ | [0.75, 0.81] | [0.74, 0.81] |
| λ_hip_ | [0.66, 0.75] | [0.71, 0.78] |
| λ_upper leg_ | [0.63, 0.69] | [0.66, 0.69] |
| λ_lower leg_ | [0.25, 0.30] | [0.31, 0.36] |
| λ_foot_ | [0.68, 0.76] | [0.73, 0.80] |
